# Hermaphroditism in Marijuana (*Cannabis sativa* L.) Inflorescences – Impact on Floral Morphology, Seed Formation, Progeny Sex Ratios, and Genetic Variation

**DOI:** 10.3389/fpls.2020.00718

**Published:** 2020-06-25

**Authors:** Zamir K. Punja, Janesse E. Holmes

**Affiliations:** Department of Biological Sciences, Simon Fraser University, Burnaby, BC, Canada

**Keywords:** self-fertilization, feminized seeds, hermaphroditic flowers, genetic diversity, transposable elements, sexual reproduction, *Cannabis sativa* L., marijuana

## Abstract

*Cannabis sativa* L. (hemp, marijuana) produces male and female inflorescences on different plants (dioecious) and therefore the plants are obligatory out-crossers. In commercial production, marijuana plants are all genetically female; male plants are destroyed as seed formation reduces flower quality. Spontaneously occurring hermaphroditic inflorescences, in which pistillate flowers are accompanied by formation of anthers, leads to undesired seed formation; the mechanism for this is poorly understood. We studied hermaphroditism in several marijuana strains with three objectives: (i) to compare the morphological features of this unique phenotype with normal male flowers; (ii) to assess pollen and seed viability from hermaphroditic flowers; and (iii) to assess the effect of hermaphroditism on progeny male:female (sex) ratios and on genetic variation using molecular methods. The morphological features of anthers, pollen production and germination in hermaphroditic flowers and in staminate inflorescences on male plants were compared using light and scanning electron microscopy. Seeds produced on hermaphroditic plants and seeds derived from cross-fertilization were germinated and seedlings were compared for gender ratios using a PCR-based assay as well as for the extent of genetic variation using six ISSR primers. Nei’s index of gene diversity and Shannon’s Information index were compared for these two populations. The morphology of anthers and pollen formation in hermaphroditic inflorescences was similar to that in staminate flowers. Seedlings from hermaphroditic seeds, and anther tissues, showed a female genetic composition while seedlings derived from cross-fertilized seeds showed a 1:1 male:female sex expression ratio. Uniquely, hermaphroditic inflorescences produced seeds which gave rise only to genetically female plants. In PCR assays, a 540 bp size fragment was present in male and female plants, while a 390 bp band was uniquely associated with male plants. Sequence analysis of these fragments revealed the presence of *Copia*-like retrotransposons within the *C. sativa* genome which may be associated with the expression of male or female phenotype. In ISSR analysis, the percentage of polymorphic loci ranged from 44 to 72% in hermaphroditic and cross-fertilized populations. Nei’s index of gene diversity and Shannon’s Information index were not statistically different for both populations. The extent of genetic variation after one generation of selfing in the progeny from hermaphroditic seed is similar to that in progeny from cross-fertilized seeds.

## Introduction

*Cannabis sativa* L. (hemp, marijuana), a member of the family Cannabaceae, is a diploid (2n = 20) outcrossing plant which produces male and female inflorescences on different plants (dioecious). Dioecy is proposed to have evolved from a hermaphrodite ancestor in angiosperms and is found in about 6% of all angiosperm plant species (Renner and Ricklefs, 1995). It has been proposed that dioecy is a basic evolutionary mechanism to ensure cross-fertilization and, as a consequence, results in maintenance of high genetic diversity and heterozygosity (Dellaporta and Calderon-Urrea, 1993; Hamrick and Godt, 1996; Ainsworth, 2000). In dioecious plants, sex determination is governed by several factors: sex-determining genes and sex chromosomes, epigenetic control by DNA methylation and microRNA’s, and physiological regulation by phytohormones (Aryal and Ming, 2014; Heikrujam et al., 2015; Bai et al., 2019). Sexual dimorphism is expressed at very early stages of organ initiation or specification, with differential expression of genes in male and female tissues (Moliterni et al., 2004). Sex determining chromosomes have been reported in 40 angiosperm species, with 34 species having the XY system which includes *C. sativa* (Ming et al., 2011; Aryal and Ming, 2014). In this species, the karyotype consists of nine autosomes and a pair of sex chromosomes (X and Y) (Sakamoto et al., 1998). Female plants are homogametic (XX) and males are heterogametic (XY), with sex determination controlled by an X-to-autosome balance system (Ming et al., 2011). The estimated size of the haploid genome of *C. sativa* is 818 Mb for female plants and 843 Mb for male plants, owing to the larger size of the Y chromosome (Sakamoto et al., 1998). The development of molecular markers linked with sex expression in hemp was described in earlier work by Sakamoto et al. (1995, 2005), Mandolino et al. (1999), and Moliterni et al. (2004). Similar studies on marijuana are described in Punja et al. (2017).

Marijuana plants are grown commercially for their psychoactive compounds, which are produced in the trichomes that develop on flower bracts in female inflorescences (Andre et al., 2016). On occasion, it has been observed that hermaphroditic inflorescences can develop spontaneously (Small, 2017). These plants produce predominantly female inflorescences, but anthers (ranging from a few to many) may develop within the leaf axils or in pistillate flower buds. These hermaphroditic inflorescences can be induced by exogenous applications of different chemicals (Ram and Jaiswal, 1970, 1972; Ram and Sett, 1981), and by environmental stresses (Rosenthal, 1991; Kaushal, 2012), suggesting that external triggers and epigenetic factors may play a role. The hermaphrodite plants are functionally monoecious due to their ability to undergo self-pollination, but the impact of self-fertilization on progeny sex ratios and on genetic variation in the subsequent progeny has not been previously studied.

There are no previously published reports which describe the morphology of hermaphroditic inflorescences in marijuana plants. In the present study, we describe the morphological features of this unique phenotype. Anther formation, pollen production and germination were studied using light and scanning electron microscopy. We also describe for the first time the effect of hermaphroditic seed formation on the resulting female:male sex ratio using a PCR-based gender identification method. We assessed the extent of genetic variation in the progeny from self-fertilized seeds and compared that to seed derived from cross-fertilization using inter-simple sequence repeats or microsatellites (ISSR) markers. This study is the first to characterize the outcome of hermaphroditism in *C. sativa*. The results have an important bearing on the utility of hermaphrodites for the production of feminized (selfed) seed in the cannabis industry.

## Materials and Methods

### Plant Growth Conditions

**FIGURE 1:**
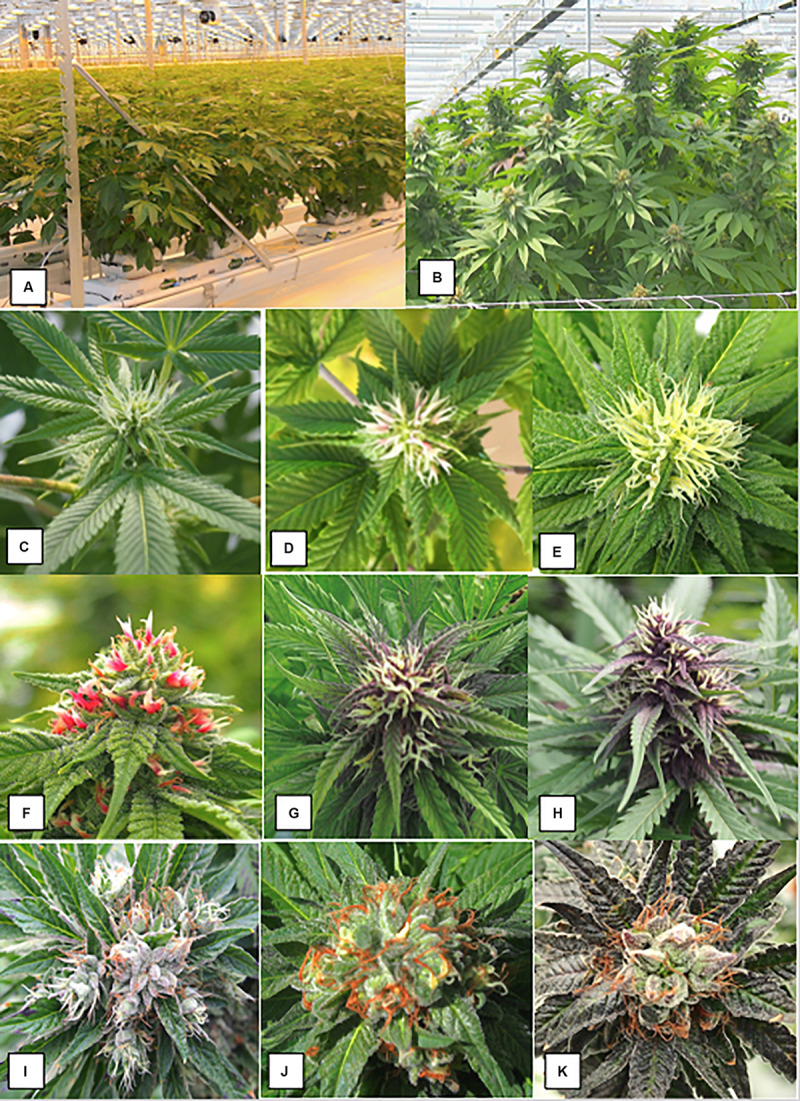
The production system for marijuana plants based on vegetatively propagated plants that are first grown under a 24 h photoperiod for 4 weeks and then switched to a 12 h dark:12 h light regime. **(A)** The plants have just been “flipped” to the reduced lighting regime following vegetative growth. **(B)** Development of large terminal inflorescence clusters in strain “Hash Plant” that extend to a 1 m height above the canopy. **(C–K)** Sequential progression of development of pistillate inflorescences on female plants of marijuana grown under indoor conditions. **(C)** Young terminal inflorescence with white hair-like stigmas. **(D)** More advanced inflorescence with yellowish-white clusters of stigmas. The stigmas are bifurcate at the tips. **(E)** Fully developed inflorescence. **(C–E)** Are of strain “Hash Plant.” **(F–H)** Are of strain “Purple God” that produces anthocyanin pigmentation. **(F)** Young terminal inflorescence with developing hair-like stigmas. **(G)** More advanced inflorescence with yellowish-white clusters of stigmas. Subtending bracts have accumulated a purple pigment. H) Fully developed inflorescence. **(I–K)** Show stages of maturation of inflorescence. Maturation is characterized by the curling and browning/reddening of the stigmas and swelling of the carpels. **(I)** Early. **(J)** Mid. **(K)** Late. This flower bud is close to harvest and the carpels have swollen.

Three strains of marijuana – “Moby Dyck,” “Space Queen,” and “Lemon Nigerian” – were cultivated in a commercial indoor growing facility according to the regulations and guidelines issued by Health Canada under an MMPR – Marihuana for Medical Purposes – license. The plants were initiated from rooted cuttings and provided with the nutrient regime for hydroponic culture as described elsewhere (Punja and Rodriguez, 2018). Lighting, temperature and growth conditions were provided in accordance with industry production standards to ensure pistillate inflorescence development (Small, 2017). The plants were first maintained in the vegetative stage for 2 weeks at a temperature range of 23–26°C with a 24 h photoperiod under a light intensity of 120 μmol m^–2^ s^–1^. They were subsequently transferred into a flowering room for an additional 6–7 weeks to induce pistillate inflorescence development under a modified photoperiod regime that consisted of reduced lighting duration and intensity (10 h photoperiod and 70 μmol ^m–2^ s^–1^ light intensity) (Figures 1A,B).

### Female Inflorescence Development

The sequential development of the female inflorescence in marijuana strains is illustrated in Figure 1. At the early stages of development, the clusters of pistils with protruding stigmas on terminal inflorescences were yellowish-white in color (Figures 1C–E). In some strains where pigmentation was a characteristic feature, the pistils and surrounding bract tissues developed a red or purple pigmentation (Figures 1F–H). With further maturation of the inflorescence, the stigmas shriveled and developed a brown-red color, while the ovules and surrounding bracts swelled and were covered by glandular trichomes that imparted a silvery-white appearance (Figures 1I–K).

### Hermaphrodite Inflorescence Development

**FIGURE 2:**
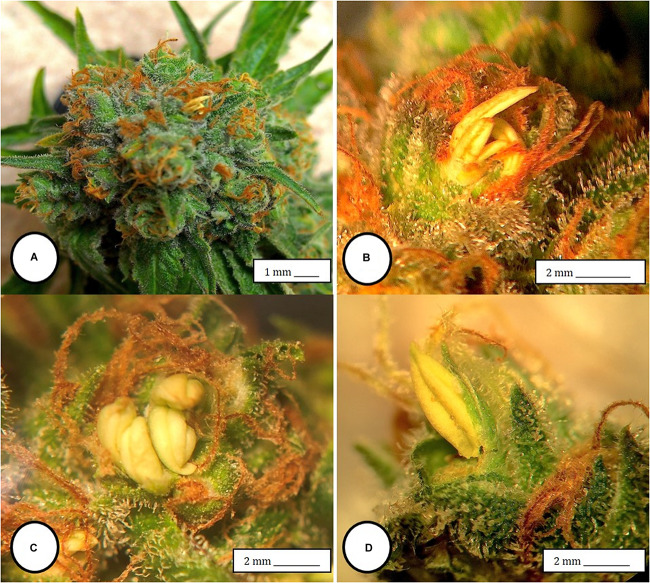
Male anthers and pollen production in hermaphroditic inflorescences of *Cannabis sativa*. **(A)** Formation of anthers within calyx tissues of female inflorescences of strain “Moby Dyck” grown under commercial conditions. **(B,C)** Close-up of clusters of anthers formed within the calyx tissues adjacent to the brown stigmas. **(D)** Pollen production and release from anthers along the line of dehiscence that appears as a longitudinal groove (stomium).

**FIGURE 3:**
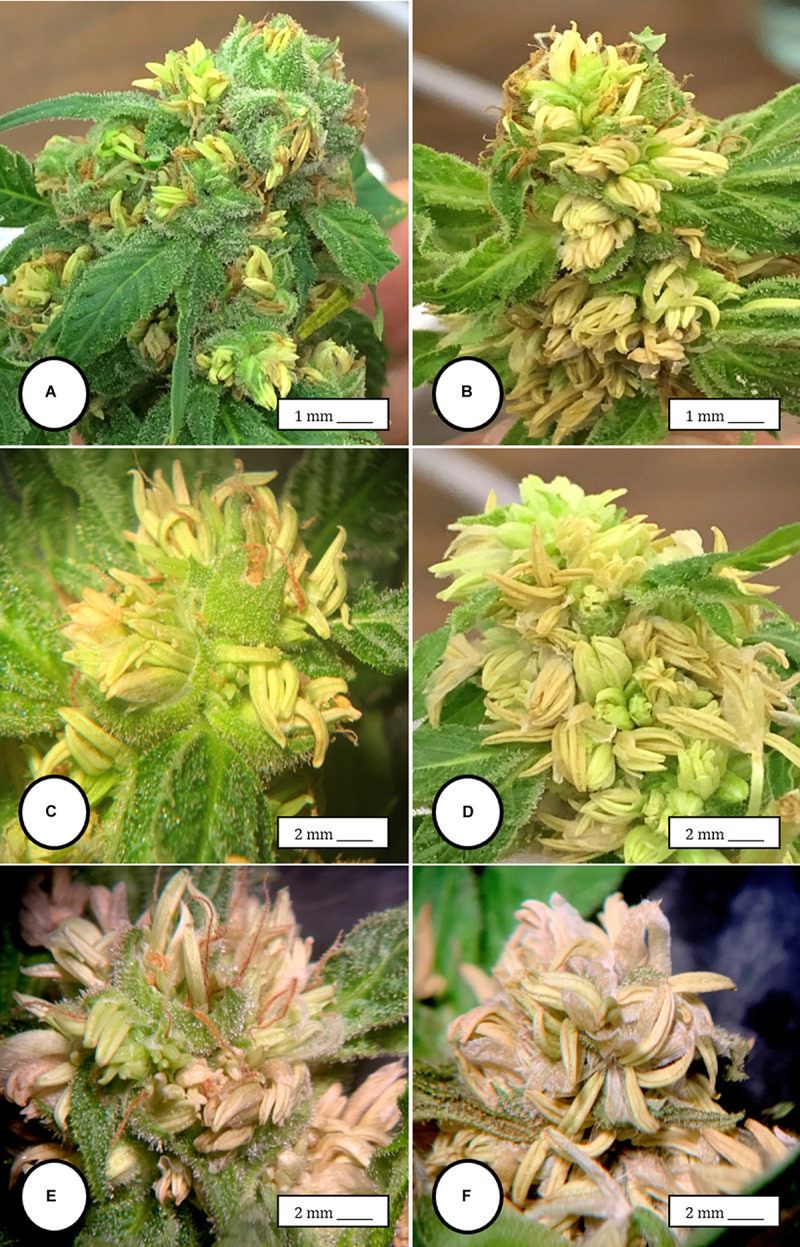
The spontaneous conversion of a female inflorescence to produce anthers. **(A)** Initial clusters of anthers forming within the calyx that normally surrounds the ovary. **(B)** Advanced stage of development of anthers in large clusters on the same plant shown in **(A,C,D)** Close-up of masses of anthers replacing the female inflorescence. **(E,F)** Mature anthers that have become dried. Figure **(F)** shows the prominent “banana-shaped” morphology. The transition from **(A–F)** occurs over 3 weeks.

During the 6–7 weeks flowering period of strains grown under commercial conditions, female inflorescences were examined at weekly intervals visually with the aid of a hand lens for the development of male anthers within the inflorescence (Figure 2). Around 1,000 plants in total were examined over the course of two repeated cycles of plant production in the study. Individual clusters of anthers appeared bright yellow and measured 2–3 mm in length (Figures 2A–D) and were formed within the bract tissues and surrounded by stigmas. In some cases, the entire female inflorescence was converted to a mass of anthers which emerged through the bracts (Figure 3). They were carefully removed with a pair of forceps, placed inside plastic petri dishes lined with moistened filter paper, and transported to the laboratory for microscopic examination. Scanning electron microscopic observations of anthers and pollen grains were made following preparation of the samples according to the procedure described by Punja et al. (2019). Pollen grains were released from anthers by suspending them in 5 ml of sterile distilled water for 2 min, from which 50 μl were plated onto 1% water agar (Bacto, Difco) and incubated at 23–25°C for 24–48 h. Percent emergence of pollen tubes was rated from 100 grains examined under an inverted compound light microscope (Zeiss). The experiment was conducted twice. In instances where seed formation was observed in the inflorescences of the three strains, they were collected at fruit maturity, counted, and set aside. Germination of a sample of 20 seeds from each strain was induced by placing them in a moist cocofibre:vermiculite (3:1, v/v) potting medium and incubating at 23–25°C for 20 days under supplemental lighting. The seedling tissues (young leaves), as well as anther tissues from hermaphroditic flowers, were used for DNA extraction (minimum of 15 seedlings per strain) as described below.

### Male Inflorescence Development

To examine the course of development of staminate flowers in genetically male plants, seeds of strains “Moby Dyck,” “Blue Deity,” and “Sweet Durga” were provided by a commercial seed producer which were produced from a controlled cross-fertilization cross. Seeds were placed in a moist cocofibre:vermiculite (3:1, v/v) potting medium and incubated at 23–25°C for 20 days under supplemental lighting as before. At least 20 seeds were germinated and 15 seedlings were obtained for each strain. The seedling tissues were used for DNA extraction as described below. The plants were grown for an additional 4 weeks under a 24 h/day photoperiod until staminate inflorescences were produced. These appeared as characteristic clusters of anthers followed by release of pollen (Figure 6). Individual clusters of anthers were carefully removed with a pair of forceps and brought to the laboratory for microscopic examination for the presence of pollen grains and for DNA extraction. In addition, scanning electron microscopic observations of anther and pollen morphology were made following preparation of the samples according to the procedure described above. To observe pollen grain germination and pollen tube growth on stigmas, pollen was manually collected from male flowers of strain “Sweet Durga” by tapping flowers gently over a piece of wax paper. The pollen was dusted onto pistillate inflorescences of strain “White Rhino” which had been collected the previous day, excised and placed in a humid chamber. A minimum of five replicate samples were included. After 72 h, tissue samples (*n* = 5) were prepared for scanning electron microscopy and germ tube production and growth on the stigmas that bore receptive papillae was determined at various magnifications from a minimum of 25 images.

### DNA Extraction, PCR, and Sequencing

DNA was extracted from leaf or anther samples using the Qiagen DNeasy^®^ Plant Mini Kit (cat. 69104, Hilden, Germany). The Quick-Start Protocol was performed with the following modifications: the tissues were ground in a microfuge tube with liquid nitrogen using a microfuge tube-sized pestle and 50 μl of Buffer AE was used to elute the DNA. Extracted DNA served as the template in PCR reactions using primers SFU1 (5′ GTGACGTAGGTAGAGTTGAA 3′) and SFU2 (5′ GTGACGTAGGCTATGAGAG 3′), the Qiagen Taq PCR Core Kit (cat. 201223), and Taq DNA Polymerase (cat. 201203). The PCR primers were designed from the MADC2 sequence from hemp (GenBank Accession No. JN426768.1) at positions 1–20 and 373–391, as per Mandolino et al., 1999). PCR conditions were as follows: one cycle of 94°C for 3 min, 40 cycles of 94°C for 30 s – 55°C for 45 s – 72°C for 2 min, one cycle of 72°C for 7 min, and hold at 4°C. The primers amplified a 540 bp sized DNA fragment in female plants, while in male plants, either two bands of 390 and 540 bp in size were produced, or just the 390 bp band was amplified (Punja et al., 2017). The 540 bp bands from the female plant samples were purified from their respective PCR reactions using NEB’s Monarch PCR & DNA Cleanup Kit (#T1030S, New England Biolabs, Toronto, Canada). The 540 and 390 bp bands from the male plant samples were separated on a 1% agarose gel then excised and purified using Qiagen’s MinElute^®^ Gel Extraction Kit (ref. 28604). The purified DNA was sent to Eurofins Genomics for sequencing in both the sense and antisense directions. Sequence files were processed and aligned, and the consensus sequences were extracted using Geneious Prime software by Biomatters Ltd. (Geneious Prime 11.1.5,^1^ Auckland, New Zeland). All sequences from the different strains representing female and male plants were aligned using the Geneious Prime multiple alignment function. These sequences were also analyzed for conserved domains using NCBI’s Conserved Domain Database (CDD). In three female strains –Moby Dick, Space Queen and Copenhagen Kush- primers S22645strt (5′CCAATAACCCTCATCCCATTCC3′) and S22645end (5′ATTTCCAAAAGTGTGCGATTCC3′) were used to amplify beyond the region of the female ∼540 bp band. The primers were designed based on a high homology BLAST result between these sequences and Scaffold 22645 of the Purple Kush Genome (canSat3) available on The Cannabis Genome Browser.^2^ Sequence files were again processed and aligned, and the consensus sequences were extracted using Geneious Prime software.

### Assessing Sequence Variation of PCR Bands From Female and Male Plants

To compare the genetic variation among bands represented by the 540 bp size following PCR, an additional 10 strains of marijuana were chosen. They ranged from landraces (“Jarilla,” “Hoa Bac Silver,” and “Brazil Amazonia”), to autoflower strains (“Northern Lights,” “Acapulco Gold,” and “Snow White”) to commercially grown strains (“CBD Therapy,” “Space Queen,” “Pennywise,” “Girl Scout Cookie,” “Copenhagen Kush”). All of the strains were genetically female, i.e., grown for production of pistillate inflorescences (female phenotype). The plants were initiated from vegetative cuttings and were grown under hydroponic conditions or in the cocofibre:vermiculite mix and provided with the nutrient and lighting conditions for commercial production by a licensed producer as described above. Both leaf tissues and DNA extracted from these strains were stored at −20°C until used. Sequence comparisons were made among the 540 bp size band in female plants of these 10 strains, between the 540 bp band in female and male plants (strains “Moby Dyck,” “Blue Deity,” and “Sweet Durga,” among the 540 bp band in male plants (where present), between the 540 and 390 bp bands in male plants, and among the 390 bp band in male plants of different strains.

### ISSR Analysis

ISSR primers UBC 807, 808, 817, 825, 834, and 842 were used to assess the extent of genetic variation. Eight samples each from leaves of strains “Moby Dyck” (ID 1 in Table 3), “Space Queen” (ID 2 in Table 3), and “Lemon Nigerian” (ID 3 in Table 3) (representing the hermaphrodite-derived population of seeds) and eight samples each from strains “Blue Deity” (ID A in Table 3), “Sweet Durga” (ID B in Table 3) and “Healer” (ID C in Table 3) (representing the female:male cross-fertilized population of seeds) were included. PCR amplifications were performed in a volume of 25 μl. Each PCR reaction contained 0.1 μM of ISSR primer, one unit of Taq DNA polymerase, 200 μM of dNTP’s, 1.5 mM MgCl2, 20 ng template DNA, and 1 × PCR buffer. Amplifications were carried out in an Applied Biosystems 2720 thermocycler programmed for 1 cycle at 94°C for 3 min for initial denaturation, followed by 40 cycles at 94°C for 30 s, 55°C for 45 s, and 72°C for 2 min and then a final extension step at 72°C for 7 min (Punja et al., 2017). After amplification, each PCR reaction was subjected to electrophoresis on a 1.5% TAE agarose gel and visualized under UV light. Gels were viewed with a Life Technologies (Thermo Fisher Scientific, Toronto, Canada) E-Gel Imager with UV-light base (cat. 4466611). The sizes of the PCR products were compared with a molecular size standard (1 kb plus) DNA ladder. Only well-separated bands of 0.1–4.0 kb size with high intensity were scored as present or absent for ISSR markers. Data were scored as “1” for the presence and “0” for the absence of DNA bands in each sample. Each set of experiments was repeated to ensure consistency of results. A total of 25 loci were analyzed. These data were used to run statistical analyses in the program POPGENE version 1.32 (Yeh and Boyle, 1997). The observed number of alleles (Na), effective number of alleles (Ne), percent polymorphic loci (PPL), Nei’s (1973) gene diversity (H), Shannon’s index (I), and gene flow estimate (Nm) values were calculated using POPGENE v1.32. Nm was also calculated according to the formula, Nm = (1 - Gst)/4Gst (Slatkin and Barton, 1989). These statistics were calculated between groups (strains) and between populations (hermaphroditic and cross-fertilized). Hardy-Weinberg equilibrium and random mating were assumed for both hermaphroditic and cross-fertilized populations. All *C. sativa* samples were assumed to be independent.

## Results

### Female Inflorescence Development

The production system for marijuana plants is based on vegetatively propagated plants that are first grown under a 24 h photoperiod for 4 weeks and then switched to a 12–14 h dark:10–12 h light regime. The plants in Figure 1A have just been “flipped” to the reduced lighting regime. Figure 1B shows development of large terminal inflorescence clusters in some strains, e.g., “Hash Plant” that extend to a 1 m height above the leaf canopy. The sequential development of the female inflorescence in several marijuana strains is shown in Figures 1C–K. At the early onset of flower development (weeks 1–2 of the flowering period), young terminal inflorescences developed white hair-like stigmas (Figure 1C). In subsequent weeks 3–4, development of yellowish-white clusters of stigmas which were bifurcate at the tips can be seen (Figures 1D,E). This stage was the most receptive to pollination (authors, unpublished observations). In red and anthocyanin-accumulating strains, stigma development was similar over this time period, and at advanced stages of inflorescence development, the yellowish-white clusters of stigmas were accompanied by red or purple pigmentation in the style tissues or subtending bracts (Figures 1F–H). Maturation of the inflorescence (weeks 5–6 of the flowering period) was characterized by the curling and browning/reddening of the stigmas and swelling of the carpels that occurred in the flowering period (Figures 1I,J). The mature inflorescence close to harvest (weeks 7–8) with collapsed stigmas and swollen carpels is shown in Figure 1K).

### Hermaphrodite Inflorescence Development

**FIGURE 4:**
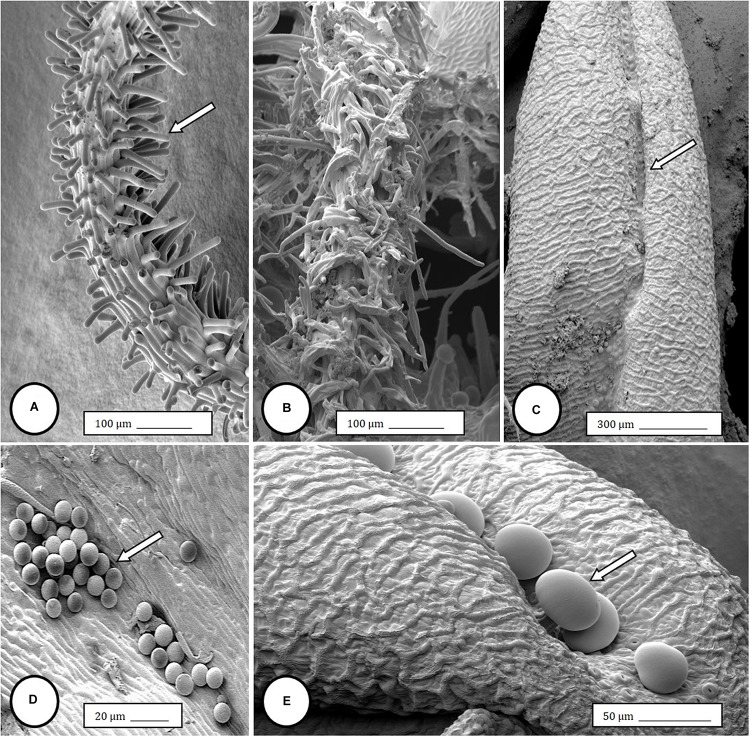
Scanning electron microscopy of the stigmas and anthers in hermaphroditic flowers of *Cannabis sativa*. **(A)** Young developing stigma with receptive papillae or stigmatic hairs (arrow). **(B)** Older stigma in which the stigmatic hairs are coiled and collapsed around a central core. **(C)** Individual anther prior to dehiscence showing an outer epidermis with the beginning of a longitudinal groove (stomium) (arrow). **(D)** Mature anther that has dehisced and revealing pollen grain release (arrow). **(E)** Enlarged view of the stomium showing formation of bulbous trichomes (arrow) forming in the groove.

**FIGURE 5:**
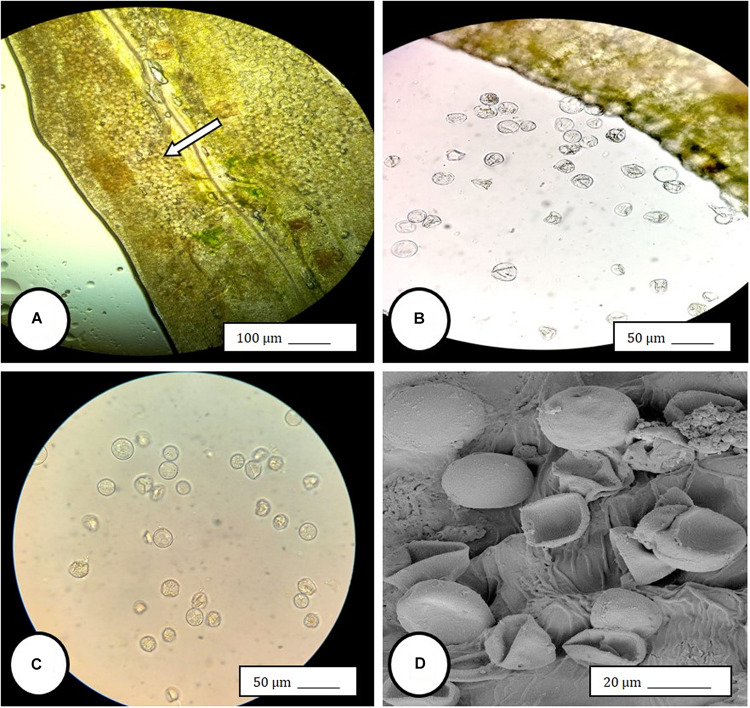
Light and scanning electron microscopic observations of anthers and pollen grains in hermaphroditic flowers of *Cannabis sativa*. **(A)** The anther wall and groove are visible and pollen grains can be seen packed within the anther pollen sacs (arrow). **(B)** Release of pollen grains into water used to mount the sample. **(C,D)** Intact and collapsed pollen grains as viewed in the light microscope **(C)** and the scanning electron microscope **(D)**.

Female inflorescences of three marijuana strains grown under commercial conditions were visually examined at weekly intervals. Beginning around week 4 of the flowering period, the appearance of individual anthers or clusters of anthers within the bract tissues adjacent to the stigmas was observed in hermaphroditic flowers at a frequency of 5–10% of the plants examined (Figures 2A–D). The anthers were visible in weeks 4–7 of the flowering period and were present until harvest. In rare instances, the entire female inflorescence was converted to large numbers of clusters of anthers (Figure 3). Scanning electron microscopic examination of the stigmas that were present in hermaphroditic flowers showed the papillae (stigmatic hairs) (Figure 4A), which in mature inflorescences originated from a central core (Figure 4B). Individual anthers that were produced in hermaphroditic inflorescences were shown to consist of an outer wall (epidermis and endothecium) with a longitudinal groove (stomium) (Figure 4C) which, upon maturity, expanded and dehisced to release pollen grains (Figure 4D). Bulbous structures presumed to be trichomes were also observed forming along the stomium of the anther (Figure 4E). When viewed under the light microscope, the anther wall and stomium could be seen and pollen grains were released into the water used to mount the sample (Figures 5A–C). Some pollen grains had collapsed when viewed under the scanning electron microscope (Figure 5D). Pollen germination was observed within 48–72 h on water agar and ranged from 10 to 30% (Figure 8A).

**FIGURE 6:**
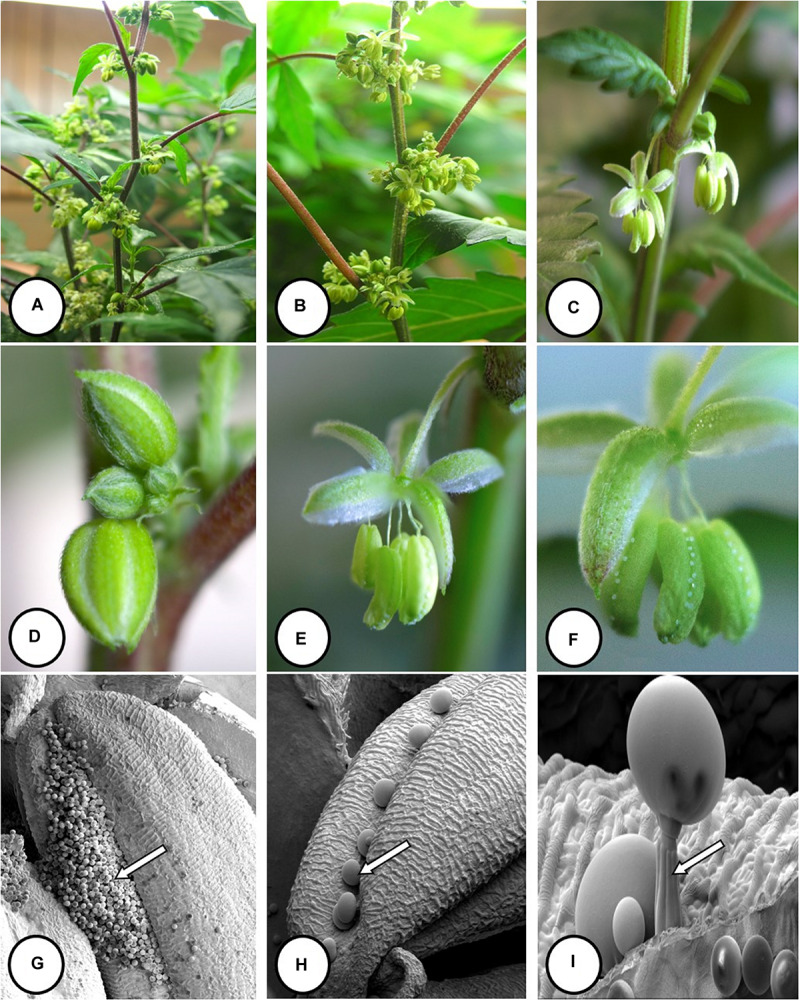
Flower and pollen development in genetically male plants of *Cannabis sativa*. **(A–C)** Male flowers formed in clusters at leaf axils. Each flower is pedicillate, with individual stalks. **(D–F)** Opening of male flowers to reveal 5 green-white tepals which expose 5 stamens each attached to a filament that dangles the anther. **(G)** Large amounts of pollen (arrow) being released through the longitudinal groove (stomium) of the anther. **(H)** Enlarged view of the stomium showing formation of bulbous trichomes (arrow) forming in the groove of the anther. **(I)** Close-up of a trichome with a short stalk (arrow). Pollen grains can be seen in the foreground.

### Male Inflorescence Development

**FIGURE 7:**
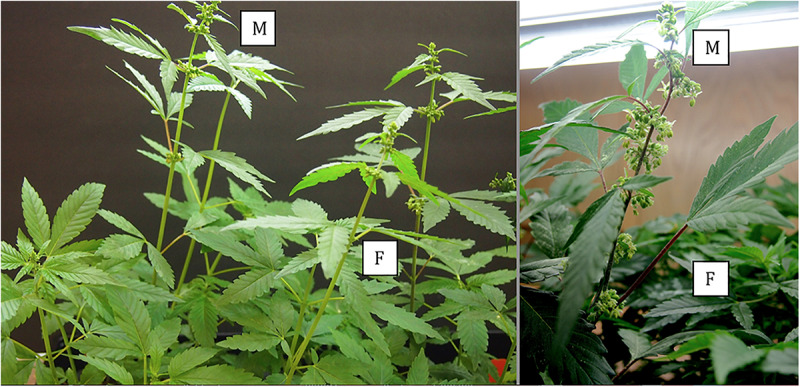
Comparative growth of male (M) and female (F) plants of *C. sativa* strain “Blue Deity,” showing the more rapid growth of male plants to achieve taller slender plants that shed pollen onto shorter slower developing female plants. Plants originated from one seed batch produced from cross-fertilization that yielded male and female plants in approximately equal ratios. Seeds were planted at the same time and grown under a 24 h photoperiod for 4 weeks.

**FIGURE 8:**
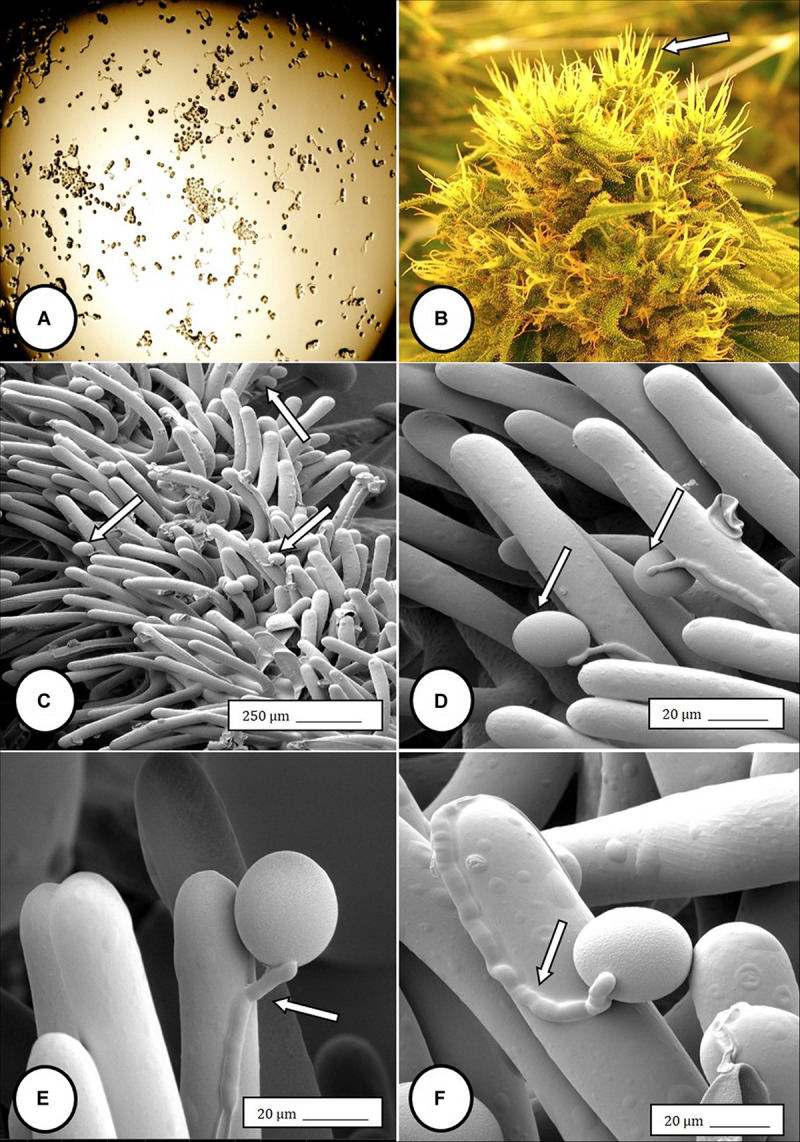
Light and scanning electron micrographs of pollen germination in *Cannabis sativa*. **(A)** Pollen germination in water after 72 h showing germ tube formation at a 20% frequency. **(B)** Female inflorescence showing protruding receptive stigmas. The flower heads were excised and pollinated *in vitro* using pollen collected from a male flower. **(C–F)** Pollen germination and germ tube development on stigmatic papillae *in situ*. Arrows show pollen grains in **(C,D)** and germ tube growth in **(E,F)**.

In genetically male plants, anthers were produced within clusters of staminate flowers that developed at leaf axils (Figures 6A–C) at around 4 weeks of age. At flower maturity in weeks 4–6, anthers dangled from individual flowers and were observed to release large amounts of pollen grains, which were deposited in yellow masses on the leaves below (Figures 6D–F, 7). Such prolific release of pollen was not observed from the hermaphrodite flowers. Scanning electron microscopic examination of the anthers produced on staminate plants showed the release of pollen grains (Figure 6G). Along the longitudinal groove or stomium, the formation of a line of bulbous trichomes (Figure 6H) that developed on a short pedicel (Figure 6I) was observed, similar to that seen in hermaphroditic flowers. When pollen from male plants was deposited onto female inflorescences (Figure 8B) and viewed at 72–96 h, various stages of pollen germination and germ tube development were observed (Figures 8C–F).

**FIGURE 9:**
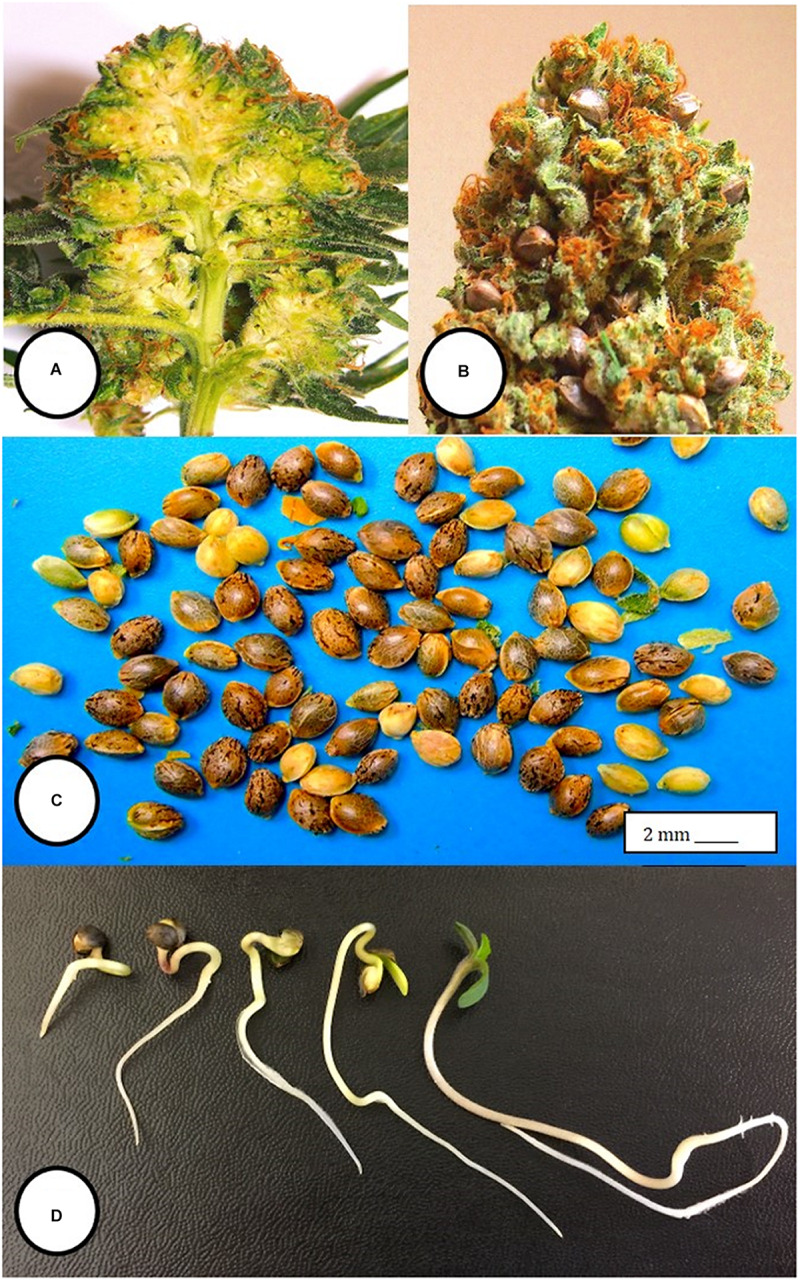
Seed formation within hermaphroditic inflorescences of *Cannabis sativa*. **(A)** Longitudinal section cut through the female inflorescence showing outer protruding stigmas and unfertilized ovules. **(B)** Seed formation within a hermaphroditic inflorescence after 3–4 weeks. Some of the calyx tissue was cut away to reveal the underlying seeds. **(C)** Seeds recovered from hermaphroditic flowers, ranging from mature (brown) to immature (yellowish-green). **(D)** Stages of seed germination after placement in a cocofibre:vermiculite potting medium and incubation for 10 days.

Within the hermaphroditic inflorescences in which anthers were found, seed set was initiated, and mature seeds were observed prior to the harvest period (Figures 9A,B). From each of 3 inflorescences bearing seeds, a total of 34, 48, and 22 seeds were obtained. The seeds were removed and placed in moist potting medium where they germinated at a rate of 90–95% within 10–14 days to produce seedlings (Figures 9C,D).

### PCR and Sequence Analysis

**FIGURE 10:**
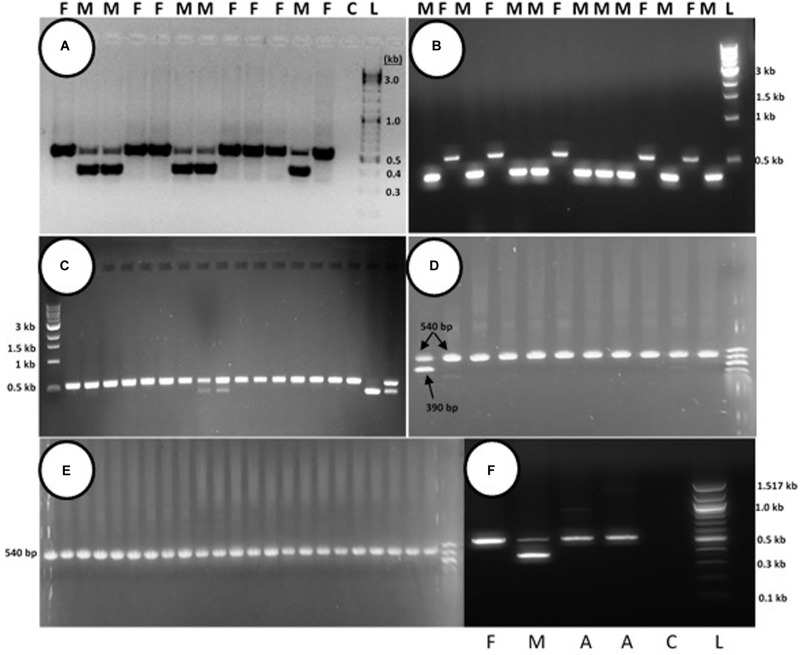
PCR analysis to identify male and female seedlings of *Cannabis sativa*. In female plants, a band of approximately 540 bp in size was observed, while in male plants, a 390 bp size band was always observed and the 540 bp band was sometimes detected. **(A,B)** Strain “Moby Dyck” and “Blue Deity” showed a 5:7 and 9:5 ratio of male (M) and female (F) plants, respectively, from seeds derived from a male:female cross. **(C)** Strain “Healer” showed a 2:14 ratio of male:female plants. **(D,E)** All female plants derived from seeds resulting from hermaphroditic flowers of strains “Moby Dyck” **(D)** and “Space Queen” **(E)**. **(F)** PCR analysis of anther tissues (A) showing female composition compared to male (M) and female (F) plants. Water control with no DNA **(C)** and 1 kb DNA ladder (NEB Quick-Load^®^) (L).

PCR analysis was used to identify specific bands which correlated with the male or female phenotype in commercial marijuana strains. Seedling tissues from strains “Moby Dyck” and “Blue Deity,” produced through cross-fertilization by a commercial seed producer, showed a band size of approximately 540 bp in female plants, while two bands (ca. 540 and 390 bp in size) or one band (390 bp), were observed in male plants. The resulting ratio of male:female plants in seeds derived from these latter strains was 5:7 and 9:5, respectively (Figures 10A,B). In a third strain “Healer,” however, which were seeds obtained from outdoor cultivation of marijuana, only two male plants were identified among 16 plants; the remaining 14 were female (Figure 10C). By comparison, seeds obtained from hermaphroditic inflorescences of strains “Moby Dyck” and “Space Queen” yielded seedlings that all showed the 540 bp band size corresponding to the female phenotype (Figures 10D,E); the male-specific 390 bp band was absent. PCR analysis of DNA isolated from anther tissues **(A)** from hermaphroditic plants showed that the banding pattern was of the 540 bp band (Figure 10F). In contrast, the banding pattern observed in staminate flower tissues showed the 390 bp band (data not shown).

### Assessing Sequence Variation of PCR Bands From Female and Male Plants

**FIGURE 11:**
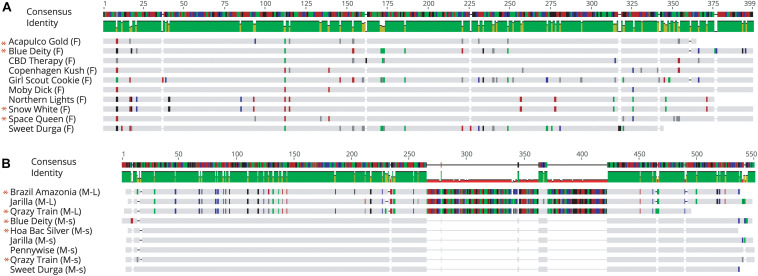
Sequence alignment of PCR fragments from female and male *Cannabis sativa* plants corresponding to the 540 bp band in female strains (F), the 540 bp band in male strains (M-L) and the 390 bp band in male strains (M-s). Only overlapping sequences were aligned for comparison. **(A)** Female sequence alignment showed 89.4–100% identity. The “*” designates sequences which have been submitted to NCBI with Accession Nos. MK093855, MK093861, and MK093858, respectively. **(B)** Male sequence alignment showed 95.1–97% identity in M-L and 97.5–100% sequence identity in M-s. There are 2 regions of DNA present in the 540 bp bands that are absent in the 390 bp male bands, indicating indels in this region. All 390 bp male sequences share this deleted region. The “*” designates sequences which have been submitted to NCBI with Accession nos. MK093856, MK093859, MK093854, MK093857, MK093862, and MK093860, respectively.

Sequence comparisons were made using Geneious Prime among the 540 bp size band in female plants of 10 different marijuana strains, between the 540 bp band in female and male plants, among the 540 bp band in male plants (where present), between the 540 and 390 bp bands in male plants, and among the 390 bp band in male plants of different strains. The 540 bp band in female plants showed sequence similarities between 89.4 and 100% among different strains (Table 1). Strain “Girl Scout Cookie” showed the lowest sequence similarity (89.4–94.1%) to all of the other strains (Table 1), particularly to the autoflower strains “Northern Lights” and “Snow White.” Strain “Moby Dyck” showed 99.3% sequence similarity to “Copenhagen Kush” (Table 1). These variations in sequences could be due either to sequence divergence over time (SNPs) or base-calling errors introduced during sequencing. In comparing the sequences of the 540 bp band present in female and male plants, the range of sequence similarity was 87.3–98.1%, with strain “GSC” showing the lowest similarity (87.3–90.6%) to the male 540 bp band sequences of three strains that were included in this study (Table 2). The 540 bp band in male plants showed 95.1–97% similarity to each other while the 390 bp band sequence in male plants had 97.5–100.0% sequence similarity (data not shown). In comparing the 540 and 390 bp bands in male plants, two regions of about 176 bp were absent within the 390 bp band, suggesting an internal deletion had occurred (Figure 11). The 540 bp band had 95% sequence similarity with a SINE MADC2 sequence (GenBank Accession No. JN426768.1) and 86% similarity with a MADC2 male-specific sequence (GenBank Accession No. JF298280.1). The 390 bp band had 100% sequence homology in the aligned overlapping region with JF298280.1 and 88% sequence homology with JN426768.1. The sequence variations among the 540 bp band present in different female strains is likely due to the presence of SNP’s detected in these strains.

**TABLE 1:** Sequence comparisons of the ∼540 bp band among 10 female *Cannabis sativa* strains.

	Acapulco Gold	Blue Deity	CBD Therapy	Copenhagen Kush	Girl Scout Cookie	Moby Dyck	Northern Lights	Snow White	Space Queen	Sweet Durga
Acapulco Gold		95.2	96.6	96.6	93.2	96.9	94.3	94.3	96.1	93.5
Blue Deity	95.2		94.6	92.8	93.9	93.3	93.0	91.4	92.3	95.9
CBD Therapy	96.6	94.6		95.5	92.8	95.6	94.2	93.7	94.8	94.3
Copenhagen Kush	96.6	92.8	95.5		90.4	99.3	95.5	95.7	97.3	92.2
Girl Scout Cookie	93.2	93.9	92.8	90.4		90.9	90.0	89.4	90.2	94.1
Moby Dyck	96.9	93.3	95.6	99.3	90.9		95.9	96.1	98.9	92.6
Northern Lights	94.3	93.0	94.2	95.5	90.0	95.9		100.0	94.0	91.6
Snow White	94.3	91.4	93.7	95.7	89.4	96.1	100.0		94.3	91.6
Space Queen	96.1	92.3	94.8	97.3	90.2	98.9	94.0	94.3		92.2
Sweet Durga	93.5	95.9	94.3	92.2	94.1	92.6	91.6	91.6	92.2	

*Percentage identity is shown.*

**TABLE 2:** Sequence comparisons of the ∼540 bp band between three male (M) and 10 female (F) *Cannabis sativa* strains.

	Brazil Amazonia (M)	Jarilla (M)	Qrazy Train (M)
Acapulco Gold (F)	96.8	92.5	95.3
Blue Deity (F)	93.2	90.5	93.4
CBD Therapy (F)	96.3	92.7	95.1
Copenhagen Kush (F)	98.1	93.8	95.2
Girl Scout Cookie (F)	90.6	87.3	89.7
Moby Dyck (F)	98.6	94.1	95.6
Northern Lights (F)	95.8	92.5	94.0
Snow White (F)	95.8	93.3	93.8
Space Queen (F)	97.4	93.1	94.7
Sweet Durga (F)	93.0	89.8	92.4

*Percentage identity is shown.*

**TABLE 3:** Mean values of statistical analyses comparing variation between cross-fertilized and hermaphroditic populations of *C. sativa*.

Population	Cross-fertilized				Hermaphroditic			
		
ID	A	B	C	A–C	1	2	3	1–3
Sample size	8	8	8	24	8	8	8	24
Na	1.6 ± 0.5	1.6 ± 0.5	1.72 ± 0.4583	1.96 ± 0.2*	1.64 ± 0.4899	1.64 ± 0.4899	1.44 ± 0.5066	1.88 ± 0.3317*
Ne	1.5145 ± 0.4495	1.4978 ± 0.4236	1.5301 ± 0.3902	1.7553 ± 0.2655*	1.4831 ± 0.3989	1.4301 ± 0.4111	1.3338 ± 0.4270	1.5799 ± 0.3403*
H	0.2739 ± 0.2325	0.2709 ± 0.2272	0.2974 ± 0.2030	0.4138 ± 0.1162*	0.2707 ± 0.2147	0.2424 ± 0.2136	0.1818 ± 0.2240	0.3330 ± 0.1675*
I	0.3886 ± 0.3273	0.3861 ± 0.3228	0.4321 ± 0.2868	0.595 ± 0.1508*	0.3916 ± 0.3061	0.3564 ± 0.3001	0.2623 ± 0.3163	0.4915 ± 0.2268*
PPL	60%	60%	72%	96%	64%	64%	44%	88%
Ht	–	–	–	0.4138 ± 0.0135	–	–	–	0.3330 ± 0.0281
Hs	–	–	–	0.2807 ± 0.0175	–	–	–	0.1326 ± 0.0215
Gst	–	–	–	0.3216	–	–	–	0.3044
Nm*	–	–	–	1.0545	–	–	–	1.1426

The observed number of alleles (Na), effective number of alleles (Ne), Nei’s (1973) gene diversity (H), Shannon’s index (I), percent polymorphic loci (PPL), and gene flow estimate (Nm) values were calculated using POPGENE v1.32. Nm was also calculated according to the formula, Nm = (1 - Gst)/4Gst (Slatkin and Barton, 1989). *Mean values for Na, Ne, H, and I for cross-fertilized and hermaphroditic populations were compared using the Student’s t-test and found not to be significantly different (*p* = 0.3169, 0.0525, 0.0583, and 0.0690, respectively, for the comparisons).

**FIGURE 12:**
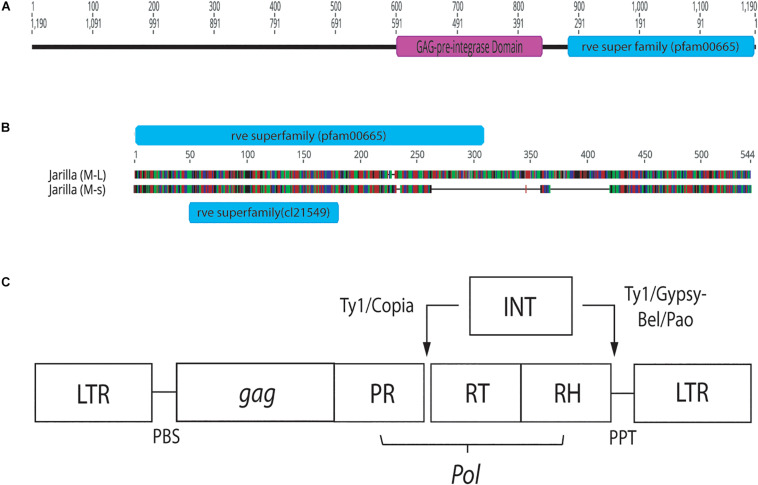
Conserved sequence domains present in representative female and male *Cannabis sativa* plants. **(A)** Conserved domains present in a female plant of strain “Space Queen” (SPQ) (Accession no. MK093858). Primers S22645strt and S22645end were used to amplify this region in the female genome beyond the ∼540 bp band produced by the GreenScreen primers. The size of the fragment, named SPQ(F)FL, is 1,190 bp. **(B)** Genious 11.1.5 pairwise alignment of representative male sequences (540 and 390 bp sizes) from strain “Jarilla.” The two indel regions total ∼178 bp. The 540 bp sequence (M-L) has the rve Superfamily pfam00665 whereas the 390 bp sequence (M-s) has the rve Superfamily core domain cl21549. **(C)** Structure of Copia and Gypsy LTR retrotransposons. This image was taken from the GyDB Gypsy Database 2.0 (http://gydb.org).

Conserved domain analysis of bands originating from female and male plants indicated the presence of an rve Superfamily integrase core domain (pfam 00665 present in the 540 bp band and pfam cl121549 in the 390 bp band size). When primers S22645strt and S22645end were used to amplify beyond the region of the female 540 bp band in three strains, a pre-integrase GAG domain (pfam13976) was found upstream of the rve Superfamily integrase core domain (Figure 12). Both domains are potential features of Class I LTR retrotransposons.

### ISSR Analysis

The six-primer set revealed a range of polymorphic bands within the populations of plants originating from hermaphroditic and cross-fertilized seeds. The percentage of polymorphic loci ranged from 44 to 64% for the hermaphroditic group of plants (*n* = 24) and 60–72% for the cross-fertilized group of plants (*n* = 24) from a total of 25 bands scored. A representative ISSR banding pattern is shown in Supplementary Figure S1. The range and mean values of statistical measures derived from data analysis for the hermaphroditic and cross-fertilized populations are presented in Table 3. To estimate the genetic variation between cross-fertilized and hermaphroditic populations and between the groups (strains) within these populations, the effective number of alleles (Ne), Nei’s (1973) gene diversity (H) and Shannon’s index values were calculated (Table 3). Hardy-Weinberg equilibrium, independence, and random mating were assumed for both hermaphroditic and cross-fertilized populations. Comparisons among the cross-fertilized groups (A,B, and C in Table 3) show overlapping mean values and standard deviations for their Ne, H, and I values. The Nei’s gene diversity (H) values for groups A, B, and C were 1.6 ± 0.5, 1.6 ± 0.5, and 1.72 ± 0.4583, respectively. This suggests that there was no observable difference in the level of genetic variation between the three cross-fertilized groups. The same observation can be made when comparing the three hermaphroditic groups (1, 2, and 3 in Table 3). With regard to mean values of the two populations, the hermaphroditic population did not differ in values of Na, Ne, H, and I when compared to the cross-fertilized population. This was confirmed by a Student’s *t*-test, in which all *p*-values were > 0.05 and therefore not significant (Table 3).

To determine if hermaphroditic populations as a whole are as genetically variable as cross-fertilized populations, the effective number of alleles (Ne), percent polymorphic loci (PPL), Nei’s (1973) gene diversity (H), and Shannon’s index values were calculated and compared (Table 3). The data showed no difference between these values (Table 3). The cross-fertilized populations had a combined Ne value of 1.7553 ± 0.2655 and the hermaphroditic populations had a combined value of 1.5799 ± 0.3403. In addition, the cross-fertilized populations had an H value of 0.4138 ± 0.1162 and the hermaphroditic populations had an H value of 0.3330 ± 0.1675. These values are not significantly different from each other. The results indicate there were no measurable differences in the population statistics used to assess genetic variation among plants after one cycle of self-fertilization as compared to cross-fertilization.

## Discussion

The spontaneous development of hermaphroditic inflorescences (pistillate flowers containing anthers) on female plants during commercial marijuana cultivation creates a problem for growers, since the resulting seed formation reduces the quality of the harvested flower (Small, 2017). The allocation of resources by the female plant to pollen production, followed by seed production, can result in disproportionately lower levels of terpenes and essential oils (by up to 56%) in the pollinated flowers compared to unfertilized female flowers (Meier and Mediavilla, 1998). Therefore, inflorescences containing seeds are of lower quality and frequently not suited for sale. Unpollinated female flowers, on the other hand, continue to expand growth of the style-stigma tissues, potentially to increase opportunities for attracting pollen (Small and Naraine, 2015), and consequently are more desirable commercially. In the present study, we observed spontaneous formation of hermaphroditic flowers on 5–10% of plants of three different strains of marijuana grown indoors under commercial conditions. In most cases, small clusters of anthers developed within certain female flowers, replacing the pistil. In rare cases (two out of 1,000 plants), the entire female inflorescence was displaced by large numbers of clusters of anthers instead of pistils (Figure 3). The factors which trigger this change in phenotype have not been extensively researched. This is due, in part, to the restrictions placed by government regulatory agencies on conducting research experiments on flowering cannabis plants (including in Canada), which reduces the opportunity to conduct the types of controlled experiments that are needed to elucidate the basis for hermaphroditism.

In earlier research, induction of hermaphroditism in marijuana plants was achieved experimentally by applications of gibberellic acid (Heslop-Harrison, 1956, 1957; Ram and Jaiswal, 1970, 1972, 1974; Galoch, 1978; Rosenthal, 1991; United Nations Office on Drugs and Crime [UNOCD], 2009). Other studies showed that male and female flower ratios in marijuana plants could be altered by applications of chemicals such as 2-chloroethanephosphonic acid, aminoethoxyvinylglycine, silver nitrate, silver thiosulfate, or cobalt chloride (Ram and Jaiswal, 1970, 1972; Ram and Sett, 1981). Silver nitrate inhibits ethylene action in plants (Kumar et al., 2009) and was reported to increase male sex expression in marijuana, cucumber and gourd plants (Atsmon and Tabbak, 1979; Ram and Sett, 1982; Stankovic and Prodanovic, 2002). In a recent study, applications of silver thiosulfate induced male flower formation on genetically female hemp plants (Lubell and Brand, 2018). These findings demonstrate that changes in growth regulator levels in treated plants can impact hermaphroditic flower formation.

Physical or chemical stresses can also have a role in inducing staminate flower development on female plants of marijuana. For example, external environmental stresses, e.g., low photoperiods and reduced temperatures in outdoor production, were reported to increase staminate flower formation (Kaushal, 2012). Some plants formed hermaphroditic flowers when female plants were exposed to extended periods of darkness early during growth or during altered photoperiods during the flowering stage, although the exact conditions were not described (Rosenthal, 1991, 2000). Such stress factors could affect internal phytohormone levels, such as auxin:gibberellin ratios (Tanimoto, 2005), which could in turn trigger hermaphroditic flower formation in marijuana plants. In *Arabidopsis* plants, auxin, gibberellin and ethylene interact with jasmonic acid (JA) to alter stamen production (Song et al., 2013, 2014). Consequently, jasmonic-acid deficient mutant *Arabidopsis* plants exhibited male sterility, with arrested stamen development and non-viable pollen (Jewell and Browse, 2016) while JA treatment restored stamen development in these mutants. In marijuana plants, environmental stress factors which enhance JA production could potentially promote hermaphroditic flower formation but this requires further study. Lability of sex expression may offer advantages in promoting seed formation in hermaphroditic plants subject to environmentally stressful conditions (Ainsworth, 2000).

In the present study, pollen germination and germ tube growth were observed in samples of hermaphrodite flowers and pollen transfer from male flowers to stigmas of female flowers showed germination *in situ* followed by germ tube growth and penetration of the stigmatic papilla. Small and Naraine (2015) and Small (2017) showed pollen grains attached to stigmatic papillae but the germination and penetration process was not described. We observed a row of bulbous trichomes forming along the stomium on the anthers in staminate flowers and in hermaphroditic flowers, confirming earlier descriptions by Potter (2009) and Small (2017) for staminate flowers. The function of these trichomes is unknown. The findings described here are the first to demonstrate viable pollen production and anther morphology in hermaphroditic flowers in marijuana.

In *Mercurialis annua*, a plant species that exhibits trioecy (co-occurrence of male, female, and hermaphrodites), male plants were observed to produce substantially more pollen than hermaphrodites (Perry et al., 2012). Our visual observations of male flowers of marijuana indicate significantly more pollen was produced and released compared to hermaphroditic flowers. These male plants released pollen over a period of 2–4 weeks; estimates are that each male flower can produce as many as 350,000 pollen grains (DeDecker, 2019). While the proportion of hermaphrodites in populations of marijuana is unknown, the frequency of seed formation within the hermaphroditic flower during indoor production is likely greater, despite the lower amounts of pollen produced, compared to a female flower dependent on wind-dispersed pollen from a male plant (indoors or outdoors). The distance over which pollen is dispersed from individual anthers in hermaphroditic flowers is probably limited to a few meters in indoor or outdoor growing facilities, compared to up to 3–5 km from male plants grown under outdoor field conditions, depending on wind speed and direction (Small and Antle, 2003). Male plants grow faster and are taller than female plants grown over the same time period, ensuring more rapid development of flowers and pollen dehiscence (Figure 7). However, the complete exclusion of male plants in indoor marijuana production suggests that the majority of seed formed would be the result of selfing. In outdoor cultivation of marijuana, where there could be several pollen sources, there is a greater likelihood of obtaining seeds that are the consequence of both self-fertilization and cross-fertilization. In Figure 10C, seeds collected from an outdoor field site showed two male plants and 14 females, contrary to the expectation of all females if they were from hermaphrodite selfing. The only explanation for the two males is that they originated from cross-fertilization with pollen from a male plant. Seeds collected from hermaphroditic flowers in indoor production in the present study all gave rise to seedlings which expressed the female genotype in a PCR-based test, compared to an approximately 1:1 ratio of male: female plants from cross-fertilized seeds. The primers amplified a 390 bp band which was present only in male marijuana plants, and a 540 bp fragment was present in male and female plants. Sequences comprising the male-specific 390 bp band were highly conserved among the 10 marijuana strains examined, and they differed from the 540 bp fragment through internal deletions of approximately 170 bp in size. Furthermore, detailed sequence comparisons of the 540 bp band showed variation due to the presence of a number of single-nucleotide polymorphisms. The internal deletion and SNP’s observed in these bands have not been previously described for *Cannabis sativa*. In the dioecious plant *Silene latifolia* (white campion), a hermaphrodite-inducing mutation was found to be localized to the Y chromosome in the gynoecium-suppression region (Miller and Kesseli, 2011). The Y chromosome plays a key role in sex determination in *S. latifolia*, and three sex-determining regions have been identified on the Y: the female suppressor region, an early stamen development region, and a late stamen development region. When hermaphrodites were used as pollen donors, the sex ratio of offspring they produced through crosses was biased toward females.

Molecular markers have been described to distinguish between male and female plants in hemp. Using RAPD markers, Sakamoto et al. (1995) observed two DNA fragments (500 and 730 bp in size) to be present in male plants and absent in female plants. The 730 bp DNA fragment was named MADC1 (male-associated DNA sequence in *Cannabis sativa*). The sequence of MADC1 did not exhibit any significant similarity to previously reported sequences. In a study by Mandolino et al. (1999), RAPD analysis revealed the association of a 400 bp band consistently with male hemp plants. Following sequence characterization of this MADC2, a low homology (54.8–59.8%) was found to retrotransposon-like elements in plants but not to MADC1. Sakamoto et al. (2005) conducted further RAPD analysis to identify additional male-specific bands in hemp (MADC3 – 771 bp in size and MADC4 – 576 bp in size) which were characterized as retrotransposable elements and reported to be present on the Y chromosome as well as on other chromosomes in male plants. Torjek et al. (2001) reported additional male-specific sequences MACS5 and MADC6 in hemp which were not homologous to any previously published sequence. Furthermore, conserved domain analysis indicated the presence of either a rve Superfamily integrase core domain alone or in conjunction with a pre-integrase GAG domain, both of which are potential features of LTR retrotransposons (Llorens et al., 2011). These previous studies suggest there are multiple sequences within the *C. sativa* genome that are associated with the male genotype, but which can also occur on other chromosomes (autosomes), many of which have similarities to transposons.

Transposable elements (TEs) have been found throughout eukaryotic genomes, including those of yeast, drosophila, rice and humans (Chénais et al., 2012). In *C. sativa*, Sakamoto et al. (2005) showed the presence of multiple Copia-like retrotransposon locations along the Y chromosome and throughout the autosomes in hemp. In the present study, a GAG pre-integrase domain was found upstream of the rve Superfamily domain in three female marijuana strains. Both domains are features of Class I LTR retrotransposons (Wicker et al., 2007; Llorens et al., 2011). According to NCBI’s Conserved Domain Database, the GAG pre-integrase domain (pfam13976) is associated with retroviral insertion elements. In addition, the placement of these domains is characteristic of the Copia Superfamily of LTR retrotransposons (Wicker et al., 2007). Therefore, the presence of Copia-like retrotransposons within the *C. sativa* genome is confirmed, but their functions or association with the expression of male or female phenotype remains to be determined.

Estimates of the degree of genetic variation (diversity) among plant populations have been obtained using isozyme markers (Cole, 2003), chloroplast DNA markers (Carvalho et al., 2019), nuclear DNA-based markers (Govindaraj et al., 2015; Bhandari et al., 2017), and single nucleotide polymorphisms (Pucholt et al., 2017; Zhang et al., 2017). In hemp, previous studies on genetic diversity assessment have utilized RAPD markers (Faeti et al., 1996; Forapani et al., 2001; Mandolino and Ranalli, 2002). Microsatellite markers, in particular, have attracted interest as a tool to assess genetic diversity in a range of plant species, including those that are diecious (Barker et al., 2003; Teixeira et al., 2009; Dering et al., 2016; Szczecińska et al., 2016; Zhai et al., 2016; Kumar and Agrawal, 2019). Measures of genetic variability are expressed as the percent of polymorphic loci (*P*), number of alleles per locus (*A*), expected and observed heterozygosity (*H_E_, H_O_*) and number of alleles per polymorphic loci (*AP*). Increased inbreeding (through selfing) and reduced frequency of polymorphic loci can result in lower levels of expected heterozygosity, particularly in small, isolated self-compatible plant species (Cole, 2003). In a dioecious out-crossing plant such as *C. sativa*, the low levels of self-pollination and extensive existing genetic variation would predict a minimal impact of hermaphroditism on genetic variation. The six-primer microsatellite set used in this study to compare the two populations originating from hermaphroditic and cross-fertilized seeds showed that the percentage of polymorphic loci, the effective number of alleles (Ne), Nei’s gene diversity (H) and Shannon’s index values had overlapping mean values and standard deviations, and were shown to not be statistically different. This indicates there was no measurable difference in the level of genetic variation between the hermaphroditic populations when compared to the cross-fertilized populations. One cycle of self-fertilization, which is the outcome from hermaphroditic seed production through selfing, may not have caused a measurable difference due to the high level of predicted heterozygosity in *C. sativa* (Small, 2017). Inbreeding can reduce the fitness of the inbred relative to outbred offspring, due to an increase of homozygous loci in the former (Charlesworth and Charlesworth, 1987). Populations that are typically outcrossing are expected to exhibit higher levels of inbreeding depression, on average, than populations that are typically selfing (Husband and Schemske, 1996). In a study involving dioecious *Mercurialis annua*, the degree of inbreeding depression (measured as seed germination, early and late plant survival, seed mass and pollen viability) was compared between outcrossed progeny and the progeny of self-fertilized feminized males (Eppley and Pannell, 2009); the findings revealed that inbreeding depression was low. Similarly, in populations of another dioecious plant, *Amaranthus cannabinus*, the effects of inbreeding on seed germination, leaf size and plant height were found to be minimal (Bram, 2002). In several dioecious plants, mechanisms to prevent inbreeding depression through selfing occur (Teixeira et al., 2009). Additional studies to determine the effects of sequential cycles of selfing on genetic variation in *C. sativa* should provide insight into whether the frequency of polymorphic loci is reduced and whether seed and plant performance measures are altered. The results from the present study suggest that one cycle of selfing to produce feminized seed (Lubell and Brand, 2018) has no measurable impact on genetic diversity in that population.

## Data Availability Statement

The datasets generated for this study can be found in the NCBI – Genbank, Accession Numbers: MK093854, MK093855, MK093856, MK093857, MK093858, MK093859, MK093860, MK093861, and MK093862.

## Author Contributions

ZP formulated the concept of the project and designed the experiments, collected the data, wrote the manuscript, and prepared the figures. JH performed the molecular experiments and data analysis and assisted with the writing. ZP and JH discussed the results and edited the manuscript.

## Conflict of Interest

The authors declare that the research was conducted in the absence of any commercial or financial relationships that could be construed as a potential conflict of interest.

## Footnotes

1 https://www.geneious.com

2 http://genome.ccbr.utoronto.ca

## Supplementary Material

FIGURE S1 ISSR analysis of seedlings obtained from plants of *Cannabis sativa* strains “Blue Deity” **(A)** and “Lemon Nigerian” **(B)**. Seeds were derived from a male:female cross in “Blue Deity” and a hermaphroditic flower in “Lemon Nigerian.” They were germinated and leaves from seedlings were used to extract DNA. Gels in **(A)** are with primer UBC 817 and in **(B)** with primer UBC 825. Ladder = 1kb (NEB Quick-Load^®^).
