# Supplementary figures and images for: Hermaphroditism in Marijuana (Cannabis sativa L.) Inflorescences – Impact on Floral Morphology, Seed Formation, Progeny Sex Ratios, and Genetic Variation
Source: Front Plant Sci. 2020 Jun 25;11:718. doi: 10.3389/fpls.2020.00718 (PMC7329997; doi:10.3389/fpls.2020.00718)

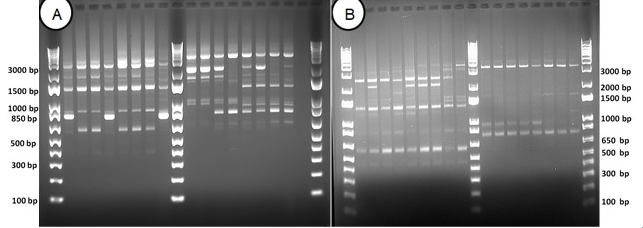

Supplement: FIGURE S1 — ISSR analysis of seedlings obtained from plants of Cannabis sativa strains “Blue Deity” (A) and “Lemon Nigerian” (B). Seeds were derived from a male:female cross in “Blue Deity” and a hermaphroditic flower in “Lemon Nigerian.” They were germinated and leaves from seedlings were used to extract DNA. Gels in (A) are with primer UBC 817 and in (B) with primer UBC 825. Ladder = 1kb (NEB Quick-Load®). [file Image_1.png]
